# A Spatial-Context Effect in Recognition Memory

**DOI:** 10.3389/fnbeh.2017.00143

**Published:** 2017-08-03

**Authors:** Daniel Pacheco, Marti Sánchez-Fibla, Armin Duff, Paul F. M. J. Verschure

**Affiliations:** ^1^Synthetic, Perceptive, Emotive and Cognitive Systems Research Group (SPECS), Department of Information and Communications Technologies, Pompeu Fabra University Barcelona, Spain; ^2^Catalan Institution for Research and Advanced Studies (ICREA) Barcelona, Spain; ^3^Institute for Bioengineering of Catalonia (IBEC), The Barcelona Institute of Science and Technology Barcelona, Spain

**Keywords:** recognition memory, spatial behavior, context effects, spatial memory and navigation, virtual reality

## Abstract

We designed a novel experiment to investigate the modulation of human recognition memory by environmental context. Human participants were asked to navigate through a four-arm Virtual Reality (VR) maze in order to find and memorize discrete items presented at specific locations in the environment. They were later on tested on their ability to recognize items as previously presented or new. By manipulating the spatial position of half of the studied items during the testing phase of our experiment, we could assess differences in performance related to the congruency of environmental information at encoding and retrieval. Our results revealed that spatial context had a significant effect on the quality of memory. In particular, we found that recognition performance was significantly better in trials in which contextual information was congruent as opposed to those in which it was different. Our results are in line with previous studies that have reported spatial-context effects in recognition memory, further characterizing their magnitude under ecologically valid experimental conditions.

## Introduction

Traditionally, the study of the dependence of human memory on environmental context has been conducted under highly constrained laboratory conditions. In general, investigations have assessed the role of context by manipulating the congruency of discrete information associated to items during learning and testing. This has typically been achieved through the use of synthetic cues of specific sensory modalities in isolation, such as colors and position of items on the screen (Murnane and Phelps, 1994; Hockley, 2008), or auditory stimuli (Geiselman and Glenny, 1977; Geiselman and Bjork, 1980). Studies with a broader definition of context have explored the role of space in recognition memory, by assessing how the congruency of the environments of encoding and retrieval affects performance in recognition tests. In such paradigms, subjects are typically presented with items to be learned in one environment, and later on tested on their ability to recognize items in the same or a different environment. Under those circumstances, consistent spatial-context effects in recognition memory have been reported in the last decades (for a review and meta analysis, see Smith and Vela, 2001).

However, key aspects of space that are known to affect memory in the animal literature have not been considered sufficiently in the environmental-context dependent memory field. In particular, theoretical work on the phenomenon of rate remapping has argued for a facilitation of memory retrieval when items are spatialized (Leutgeb et al., 2007; Rennó-Costa et al., 2010). Indeed, the retrieval of items associated with specific locations in space could benefit from pre-existing connections between hippocampal place cells depicting spatial trajectories (Lisman, 2015; Silva et al., 2015). Yet in traditional environmental-context dependent memory experiments, items have not been deployed spatially, but rather presented in a compressed form. Even in setups where environments of learning and testing are changed, stimuli are typically presented in a unique spatial location (i.e., a list of words to be read in or visual stimuli presented on a computer screen).

On the other hand, it has been shown that spatial memory in the mammalian brain is highly dependent on movement and action, being driven by a path integration signal (Rennó-Costa et al., 2010; Giocomo et al., 2011; Lu et al., 2013). Indeed, spatial information encoded by hippocampal place cells is significantly reduced when movement is restricted (Song et al., 2005; Chen et al., 2013). Yet, in previous investigations on context-dependent memory, participant’s movements during encoding and retrieval has been constrained or uncontrolled (Smith and Vela, 2001). This contrast with the long tradition of experiments in the human navigation literature where the modulation of spatial learning by spatial behavior has been explored (Chrastil and Warren, 2013). While several of these studies have been conducted in outdoor setups, others have employed Desktop Virtual Reality (VR; Wilson et al., 1997; Christou and Bülthoff, 1999; Gaunet et al., 2001; Carassa et al., 2002; for a review, see Chrastil and Warren, 2012). Although stationary VR does not assess the contribution of proprioceptive or vestibular information, it has a number of advantages that make it a valid tool for the study of human navigation. First, it captures information related to volition and cognitive decision making on the routes to take during way finding (Chrastil and Warren, 2012). Second, it has been shown to activate navigational systems of the brain including Place Cells in the hippocampus (Ekstrom et al., 2003), and Grid Cells in the Medial Entorhinal Cortex (MEC, Jacobs et al., 2013). Third, stationary VR allows the flexible manipulation of independent variables related to the configuration of space that would be otherwise impossible to handle (Tarr and Warren, 2002).

Grounded in the animal literature and previous theoretical work (Rennó-Costa et al., 2010), we aimed to test spatial-context effects in recognition memory under ecologically valid experimental conditions. We created a novel setup in which we associated discrete items to unique locations in a virtual maze. Following a within-groups methodology, we required the participants of our experiment to navigate the maze in order to find and memorize discrete images. We later on tested their ability to identify, from a new set of items, those previously presented from those that were new. Critically, we changed the contextual information associated with part of the old items during the retrieval phase of our experiment, in order to evaluate recognition memory for stimuli in congruent and incongruent spatial-context conditions.

Given the spatial context effects in recognition memory previously discussed, and the fact that we generated the conditions that are known to modulate memory in the hippocampus and the MEC, we predicted to observe a spatial context effect in our VR-based, spatial recognition test. In particular, we hypothesized to find better recognition performance and shorter decision times for items encoded in the congruent spatial context condition as compared to the incongruent one.

## Materials and Methods

### Participants

Participants were 33 young adults (20 male, mean age 23.78 ± 3.91 years) recruited from Universitat Pompeu Fabra’s (Barcelona, Spain) student community. All participants were explained about the procedures by the experimenter and provided informed consent to participate in the study. The protocol was approved by the local Ethical Committee “Clinical Research Ethical Committee (CEIC) Parc de Salut Mar” (Barcelona, Spain).

### Procedure

**Figure 1:**
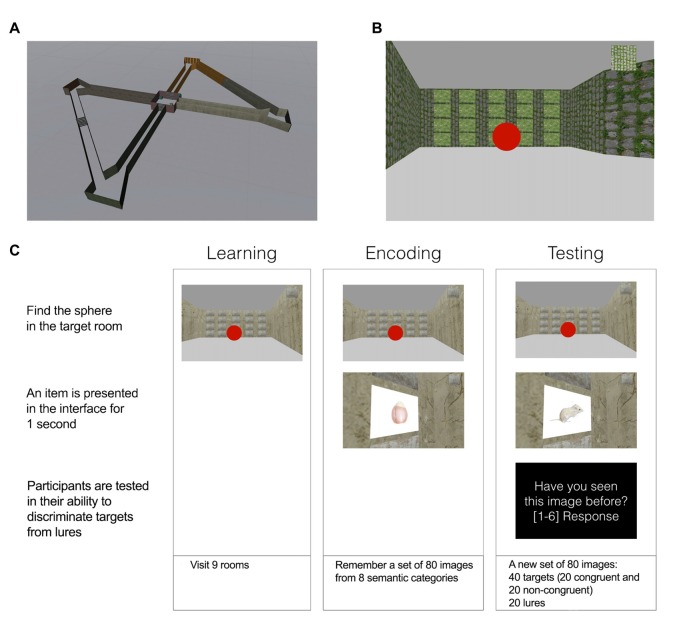
Task description. **(A)** A view showing the spatial layout of the maze. Four satellite rooms are connected to a central one. **(B)** Twenty items are positioned in one of the walls in each room forming a 5 × 4 matrix. The target room at each trial is indicated in the top right of the user interface (UI) with a texture. **(C)** Schematic showing a trial in each of the phases of the experiment.

The experiment was organized in three main blocks: learning, encoding and retrieval. In all blocks, participants were asked to navigate a virtual maze comprising a central and four satellite rooms (Figure 1A). Each room had a unique visual texture on its walls (i.e., concrete, stone, brick and wood) and was connected to a second room and to the center room. During all blocks participants had to perform a navigation task, which consisted of finding a target room (indicated in the user interface, UI, Figure 1B) and memorize (encoding block) or recognize (testing block) a discrete stimulus. A trial was defined as the action of reaching the room and memorize/recognize the correspondent stimulus. The starting position for the first trial in each block was set to the central room for all participants. The sequence of rooms to visit was randomized. In all blocks participants were instructed to perform the task until they visited all images. Participants experienced the 3D world sitting in front of a 32″ computer screen and interacted with the application using the keyboard and the mouse. The VR application was created using the Unity3D game engine (Unity Technologies, San Francisco, CA, USA).

The experiment evolved over three blocks (Figure 1C).

*Learning block*. The objective of the learning block was for the user to gain spatial knowledge of the maze before the start of memory encoding and to help participants to familiarize themselves with the interface. Before starting navigation, subjects were shown a map of the environment and explained the characteristics of the maze. During navigation, they were asked to reach the target room indicated in the UI at each trial, until they completed 10 rooms.

*Encoding block*. In the encoding block, subjects had to memorize 80 images that were encountered during navigation. Images were located in one of the walls of each room in a 5 × 4 matrix (20 images per room). The matrix was designed so that each item would be associated with a unique spatial context in the environment, determined by its location in the maze (room) and its position on the wall. When the target room was reached at each trial, the correspondent stimulus was presented for 1 s, and then made invisible for the rest of the block.

*Testing block*. In the testing block subjects were asked to navigate again and visit a new set of 80 images (20 per room), from which half have been seen previously and half were new. As in the encoding phase, after reaching the target room at each trial, an item was revealed for 1 s. After stimulus presentation, subjects were asked to indicate whether they had seen the image in the previous block of the experiment using a 6 points confidence scale—from 1 (Sure unfamiliar) to 6 (Sure familiar) as in a traditional recognition memory experiment (Squire et al., 2007). The confidence question was presented in the UI and remained visible until participants reported their answers.

Two categories of items were distinguished: those presented in the same room and position on the wall matrix during the encoding and retrieval phases of the experiment (congruent items), and those presented in a different room and position on the wall (incongruent items).

All items (targets and lures) were extracted from the same pool of images belonging a dataset available in Moreno-Martínez and Montoro (2012). From the 360 objects in the dataset, a smaller pool of 160 items was selected from eight semantic categories (food, furniture, human body, musical instruments, buildings, tools, clothes and animals). For each subject separately, a subset of 80 images was selected randomly from this pool of 160 images, and assigned (again randomly) to specific conditions. In total, 20 images were assigned to the congruent condition and 20 to the incongruent condition. Eighty images were assigned to the “new” condition, from which 40 were presented during encoding and not shown at retrieval, and 40 were presented at retrieval but not shown during the encoding phase of the experiment.

Participants were not instructed to optimize their spatial behavior in order to find the shortest path connecting two rooms, but were informed about the spatial layout of the environment during the learning phase.

### Dependent Variables

Three dependent variables were measured in the experiment.

**Recognition accuracy:** number of correct answers divided by the total number of trials in a correspondent condition.

**Decision Time (DT):** time elapsed from question onset until participant’s responses. We only considered correct responses and excluded trials in which it was more than 2.5 SD away from the mean for each participant separately.

**Navigation Optimality:** ratio between the shortest possible trajectory length connecting initial and target rooms at each trial and the actual trajectory length of that trial. The measurement ranges from 0 (if a subject never reaches the target room) to 1 (shortest trajectory length). Given the shape of the environment, only one possible combination of corridors connecting two rooms was the correct choice to achieve optimal performance. This measure was created to evaluate how well participants learnt the maze before the testing phase of the experiment.

A repeated measures ANOVA model was built to characterize the relationship between familiarity (novel/familiar), and confidence (high/low) with recognition performance. A second ANOVA model was constructed to quantify the influence of elapsed time (short/long) and old item condition (congruent-incongruent) on recognition performance. For the analysis of DTs we used a one-way ANOVA with factor item condition (old congruent—old incongruent—new). Pairwise *post hoc* comparisons were performed using paired *t*-tests. All reported *post hoc* tests are corrected for multiple comparisons using the Bonferroni method.

## Results

### Overall Results

We first aimed to evaluate if our VR based recognition test could capture the dynamics of recognition memory previously identified in traditional laboratory setups (Squire et al., 2007; Wixted, 2007). We conducted a Receiver Operating Characteristic (ROC) analysis for each participant, to assess how well they could discriminate targets and lures at different levels of confidence. A ROC is simply a plot of the hit rate (old items are correctly identified as old) as a function of the false-alarm rate (new items are incorrectly identified as old) at different levels of confidence (Squire et al., 2007).

**Figure 2:**
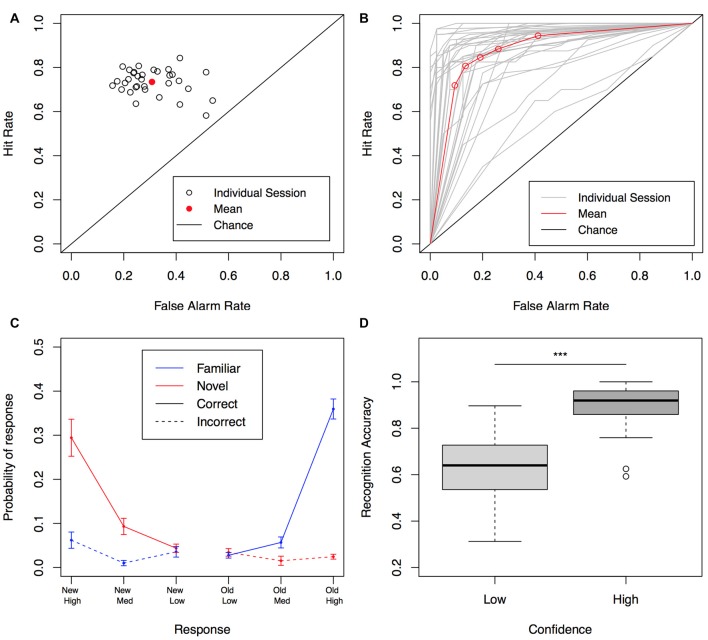
Recognition performance. **(A)** Performance as a function of proportion of trials correctly and incorrectly identified. Each point is one session (*n* = 33); red point indicates the mean performance. **(B)** Behavioral Receiver Operating Characteristic (ROC) curve for individual sessions (gray) and average (red). Each data point is a different confidence level. **(C)** Response probabilities for familiar and novel, correct and incorrect items. Error bars represent ± SE across subjects. **(D)** Normalized recognition accuracy was significantly higher in trials recognized with high confidence compared to low confidence trials. ***P ≤ 0.001. *P* values are corrected for multiple comparisons.

All participants performed above chance levels (0.5 for Hits and False Alarms, along the diagonal in Figures 2A,B) at different levels of confidence. The average area under the curve (AUC) was 0.86 ± 0.01 (SE, Figure 2B). As expected for declarative memories (Kahana, 2012), the z-ROC curve was asymmetrical along the chance diagonal (mean z-ROC slope = 0.86), indicating a greater variance in the distribution of the old items memory strength (Squire et al., 2007).

Participants responded in general with high confidence (74.9% of the trials, SE = 4.6%); medium and low confidence were assigned less frequently and in similar proportions (*M* = 17.4%, SE = 2.3% and *M* = 13.3%, SE = 1.7% respectively, Figure 2C). Due to this unequal distribution, we pooled data from trials with intermediate and low confidence ratings for a comparison between high and low confidence with an equivalent number of samples. Normalized accuracy was significantly better in the high confidence group (0.89 ± 0.01 vs. 0.64 ± 0.02; main effect of confidence, *F*_(1,32)_ = 97.9, *P* < 0.01, *η*^2^ = 0.76). *Post hoc* tests revealed that indeed performance for items retrieved with high confidence was significantly higher than low confidence trials *t*_(32)_ = 9.98, *p* < 0.01, *d* = 1.73; Figure 2D.

In general, these results are consistent with a recent report that measured performance, confidence and decision times (DTs) in a traditional recognition memory experiment (Rutishauser et al., 2015), suggesting that the main features of recognition memory were well captured by our spatial test.

### Spatial Behavior

**Figure 3:**
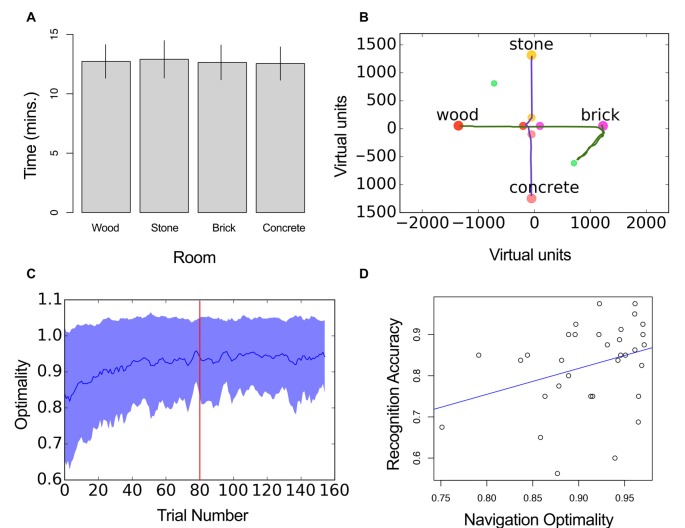
Navigation performance. **(A)** Mean time of all participants in each room. Error bars represent ±SD across subjects. **(B)** Example trajectories from the same participant indicating different optimality values (green = 0.97, pink = 0.60). **(C)** Optimality in navigation as a function of trial number. Trial 80 marks the start of the testing block. Blue line represents mean optimality and shaded area the standard deviation. **(D)** Navigation optimality as a function of recognition accuracy. Blue line shows the least square fit.

The analysis of spatial behavior revealed that all four satellite rooms were occupied an equivalent amount of time across participants (Wood: *M*: 12.72 min, SD = 1.41 min, Stone: *M* = 12.9 min, SD = 1.57 min, Brick: *M* = 12.64 min, SD = 1.46 min, Concrete: *M* = 12.54 min, SD = 1.4 min, *F*_(3,96)_ = 2.58, *P* = 0.06, *η*^2^ = 0.07, Figure 3A). Two example trajectories are shown in Figure 3B.

Participant’s navigation was close to optimal after the learning session, although some learning also took place during the first trials of the encoding block (Figure 3C). Mean optimality was higher in the testing block (*M* = 0.91, SE = 0.01) compared to the encoding block (*M* = 0.87, SE = 0.009, *t*_(32)_ = 7.76, *p* < 0.01, *d* = 1.35; Figure 3C). The individual data revealed the different profile of responses observed across participants (Supplementary Figure S1).

We aimed to assess the statistical dependency of navigation optimality and recognition accuracy. For this we calculated a mean optimality value for each session and participant. Neither optimality or recognition accuracy were normally distributed (Shapiro Wilk’s normality test, *p* < 0.05). Therefore, we used the Spearman’s rank correlation coefficient. Results indicated that optimality in navigation and overall performance in the recognition test were positively correlated, *r*_(3753)_ = 0.37, *p* = 0.03, Figure 3D.

We next assessed the relationship between the two variables by dividing subjects into those who scored high in navigation optimality (>median of all subjects), and those that performed low (<median). Recognition accuracy was not significantly different in the two groups *t*_(29)_ = 1.5, *p* = 0.14, *d* = 0.53.

### Spatial Context Modulates Recognition Performance

The main objective of this research was to assess differences in performance related to the congruency of spatial context of items at encoding and retrieval. Target items in the retrieval block were presented in the same room and position on the wall with respect to their location when encoding took place (congruent trials), or in a different room and different position on the wall (non-congruent trials).

**Figure 4:**
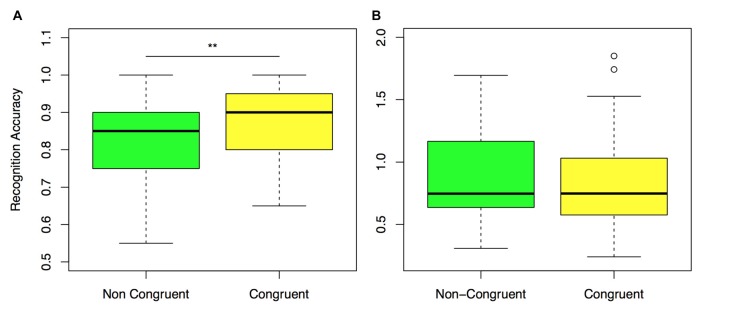
Recognition performance for congruent and non-congruent items. **(A)** Normalized accuracy in non-congruent and congruent trials. **(B)** Decision Time (DT) for non-congruent and congruent trials. **P ≤ 0.01. *P* values are corrected for multiple comparisons.

A repeated measures ANOVA model revealed a main effect of item condition (old congruent–old incongruent) in recognition performance, *F*_(1,32)_ = 7.77, *P* < 0.01, *η*^2^ = 0.19. *Post hoc* analysis confirmed that accuracy in recognition was better for congruent compared to non-congruent trials (Congruent: *M* = 0.87, SE = 0.01, Non-congruent: *M* = 0.81, SE = 0.02, *t*_(32)_ = 2.9, *p* = 0.01, *d* = 0.51, Figure 4A).

Given that the testing block included a recognition test at each trial, mean duration was significantly higher in the testing as compared to the encoding block (Encoding: *M* = 28.67, SD = 3.75, Testing: *M* = 33.45, SD = 7.51, *t*_(32)_ = 4.1, *p* < 0.01, *d* = 0.71). Since subjects navigated with different levels of optimality and that the order of presentation of items was randomized, the target item’s elapsed time between encoding and retrieval was different at each trial. To assess the influence of this factor in recognition performance, we split trials into those with short and long elapsed time (below and above the median for each subject) and included this factor in our ANOVA model. We found a main effect of elapsed time on recognition performance (*F*_(1,32)_ = 4.89, *P* < 0.05, *η*^2^ = 0.13). This effect did not reach significance after Bonferroni correction in the *Post hoc* Analysis (Short: *M* = 0.86, SE = 0.01, Long: *M* = 0.82, SE = 0.01, *t*_(32)_ = 2.23, *p* = 0.09, *d* = 0.38). No interaction between elapsed time and item condition was found (*F*_(1,32)_ = 0.01, *P* < 0.89, *η*^2^ = 0.0005).

We next checked whether the observed differences in performance for congruent and incongruent items were confirmed in DT. Analysis showed a significant main effect of condition *F*_(2,64)_ = 10.88, *p* < 0.01. *Post hoc* analysis revealed that although DT was shorter for congruent items (*M* = 0.87, SE = 0.09), compared to non-congruent (*M* = 0.98, SE = 0.11), this difference did not reach significance after Bonferroni correction, *t*_(32)_ = 2.9, *p* = 0.05, *d* = 0.4 (Figure 4B). New items, on the other hand, were recognized with significantly larger DTs than old congruent and old incongruent trials (new: *M* = 1.2, SE = 0.11), compared to old congruent *t*_(32)_ = 4.56, *p* < 0.01, *d* = 0.79; compared to old incongruent *t*_(32)_ = 7.14, *p* < 0.01, *d* = 1.24.

## Discussion

According to the encoding specificity principle (Tulving and Thomson, 1973), human memory is improved when information available at encoding is also available at retrieval. Previous research has shown that the principle is valid in recognition memory (Smith and Vela, 2001), although in highly constrained experimental setups. Here, we approached the problem from the perspective of space and human spatial behavior. Using VR, we could generate encoding and retrieval conditions that are more likely to occur in the real world, and that are known to modulate memory in rodents (Rennó-Costa et al., 2010; Lu et al., 2013). In our setup, each item to be learned was associated with a unique spatial context, and active navigation was required to reach the items at encoding and retrieval. This allowed us to create a strong contextual association for each item, which included spatial and navigational aspects.

Our results confirm a significant modulation of recognition memory by incidental context. Recognition accuracy was significantly higher for items in which contextual information was congruent compared to those in which it was different. Furthermore, we identified a tendency for congruent items to be recognized in shorter DTs as compared to incongruent ones, although this difference did not reach significance after Bonferroni correction (*p* = 0.05).

These findings contribute to a long tradition of investigations that have explored the modulation of recognition memory by environmental context in experimental psychology. Indeed, early studies have reported mixed results on the effects of spatial context in recognition i.e., inexistent (Fernandez and Glenberg, 1985) or small (Smith, 1985; Murnane and Phelps, 1994). Our data confirms the current view that such effects exist (Smith and Vela, 2001), and we further quantify that recognition accuracy was 6% higher for items in which spatial position at encoding and retrieval was congruent compared to that of trials in which it was different (a bigger effect than the 2% reported for instance in Smith, 1985, for lists of words).

On the other hand, the analysis of trajectory data revealed that on average subjects learned the maze throughout the experiment, which is reflected in the increased navigation performance during the testing as compared to the encoding block. Interestingly, we observed that navigation optimality and recognition accuracy were positively correlated. However, the observed variability in the individual data indicated that navigation optimality was differently liked to performance in the memory test across subjects (Supplementary Figure S1). For instance, subject 15 navigated optimally (Mean optimality = 0.96) but had an overall recognition accuracy of 0.68, whereas subject 8 navigated similarly well (Mean optimality = 0.98), with an overall memory of 0.975. The high inter-subject variability and the fact that some subjects performed poorly in the last trials of the encoding block suggests that participants used different strategies to navigate and did not learn the maze equally. Although the positive correlation we observe is consistent with a critical role of space representation in the association of items with their context (Nadel, 2008), the relationship of spatial learning and memory for spatialized items will require further investigation.

A final remark related to the spatial behavior analyses is that navigation performance affected the time elapsed between the encoding and retrieval blocks of the experiment. At an item level, the time elapsed between these two moments was dependent on spatial performance and on the order of presentation of stimuli, which was randomized. We controlled for this potential confound variable in our analyses and found that time elapsed did not significantly affected memory and did not interact with the modulation of recognition performance by spatial-context previously discussed.

In terms of overall accuracy, our results are consistent with several studies that have characterized human behavior in recognition memory experiments (Squire et al., 2007; Rutishauser et al., 2015). This can for instance be appreciated in the asymmetrical shape of the ROCs, the distribution of AUCs, the enhanced performance in high confidence trials as compared to low confidence ones, or in the faster DTs for familiar items as compared to new ones (Squire et al., 2007; Rutishauser et al., 2015).

Moreover, our results suggest that VR can be a powerful tool to investigate the modulation of memory by incidental context. Previous studies have questioned the validity of virtual environments for the study of spatial behavior and particularly route memory (van der Ham et al., 2015). Indeed, disembodied navigation does not capture key factors of navigation that affect memory, such as proprioceptive or vestibular information (Chrastil and Warren, 2012). Nonetheless, it is likely that the effects we observe are not related solely to the visual information associated with the items, but to a broader context which includes spatial and navigational aspects. It has been shown that the brain systems for navigation including Grid and Place cells activate during stationary VR navigation (Ekstrom et al., 2003; Jacobs et al., 2013). Moreover, place cells that fire when specific items are encoded during navigation in Desktop VR also discharge when these items are retrieved in free recall (Miller et al., 2013). We speculate that the activity of place cells representing space might have contributed to a stronger context effect in our setup, even in the absence of real movement. Indeed, even if subjects were not actually moving when the stimulus was presented, each item was shown in a specific place, i.e., a room, probably encoded by place cells in the hippocampus. The specific contribution of embodiment in the modulation of memory will remain to be determined in future experiments.

In future research, we also aim to investigate the neural signatures of the behavioral effect we observe in the human hippocampus, a key structure in the binding of items and context (Bird and Burgess, 2008; Nadel, 2008; Dede et al., 2013). Indeed, previous research conducted with intracranial recordings in humans has shown increases in hippocampal gamma power for associative vs. non-associative recognition in a non-spatial setup (Staresina et al., 2016). Moreover, neurons in the hippocampal formation have been reported to encode several components of recognition tasks, such as object categories, novelty/familiarity and confidence (Rutishauser et al., 2015). Hippocampal cells have also been linked to the encoding of recollection and familiarity—both key cognitive processes that are thought to underlie recognition memory (Merkow et al., 2015).

In summary, by extending the study of recognition memory to the domain of spatial behavior, we report for the first time human behavioral data expressing the link between space and recognition memory performance. This is a novel finding that extends our current understanding of recognition memory, and could be used in the design of novel educational paradigms (Pacheco et al., 2014).

## Author Contributions

The original hypothesis was advanced by PFMJV and DP, MS-F, AD and PFMJV contributed to the concept and design of the experiment. Testing and data collection were performed by DP. Data analysis and interpretation were conducted by DP and MS-F, DP and PFMJV wrote the manuscript. All authors approved the final version of the manuscript for submission.

## Conflict of Interest Statement

The authors declare that the research was conducted in the absence of any commercial or financial relationships that could be construed as a potential conflict of interest.
