# Supplementary material for: A Spatial-Context Effect in Recognition Memory
Source: Front Behav Neurosci. 2017 Aug 3;11:143. doi: 10.3389/fnbeh.2017.00143 (PMC5541067; doi:10.3389/fnbeh.2017.00143)
Supplement: Supplementary file 1 [file Data_Sheet_1.docx]

**SUPPLEMENTARY INFORMATION**

**
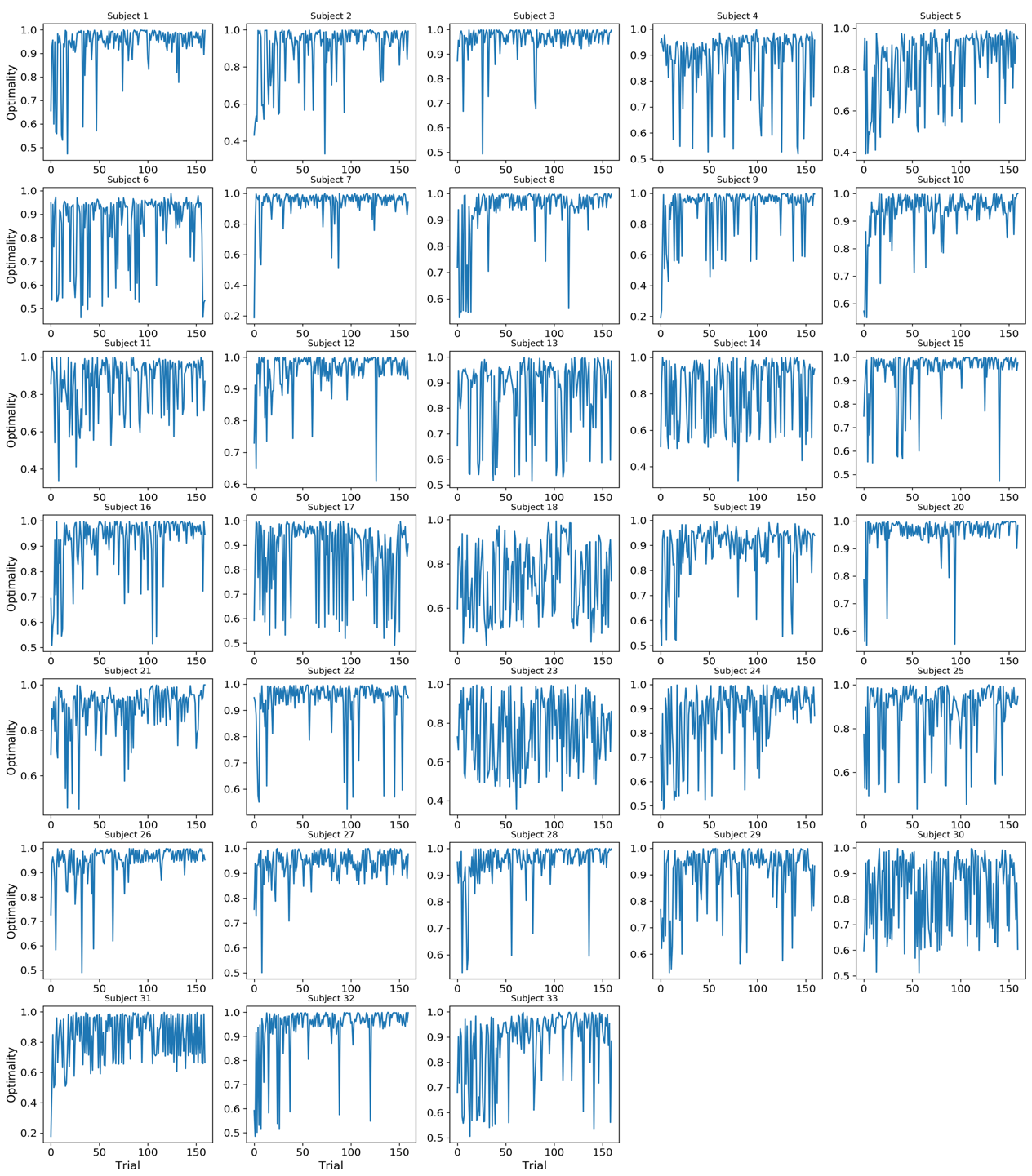
**

**Figure S1** Navigation Optimality per subject. Each plot is the optimality curve (y axis) plotted as a function of trial number (x axis). The average data can be found in Figure 3a. The relation of optimality and recognition accuracy is shown in Figure 3d.
